# New spinosin derivatives from the seeds of *Ziziphus mauritiana*

**DOI:** 10.1007/s13659-013-0028-5

**Published:** 2013-05-07

**Authors:** Bin Wang, Hong-Tao Zhu, Dong Wang, Chong-Ren Yang, Min Xu, Ying-Jun Zhang

**Affiliations:** 128 State Key Laboratory of Phytochemistry and Plant Resources in West China, Kunming Institute of Botany, Chinese Academy of Sciences, Kunming, 650201 China; 228 University of Chinese Academy of Sciences, Beijing, 100049 China

**Keywords:** *Ziziphus mauritiana*, Rhamnaceae, seeds, spinosin derivatives, jujuboside A, acetylcholinesterase inhibitory

## Abstract

Three new acylated flavonoid *C*-glycosides, 6‴-(-)-phaseoylspinosin (**1**), 6‴-(3″″,4″″,5″″-trimethoxyl)-(*E*)-cinnamoylspinosin (**2**), and 6‴-(4″″-*O*-*β*-D-gluco-pyranosyl)-benzoylspinosin (**3**), were isolated from the seeds of *Ziziphus mauritiana* (Rhamnaceae). A further 19 known compounds including eight spinosin analogues (**4–11**) were also isolated. Their structures were elucidated by means of spectroscopic analysis and chemical method. Among spinosin derivatives **1, 2, 4, 7, 8**, and triterpenoid saponin **14**, jujuboside A (**14**) displayed moderate acetylcholinesterase (AchE) inhibitory activity with an inhibition value of 46.2% at a concentration of 1 µM.

**Electronic Supplementary Material:**

Supplementary material is available for this article at 10.1007/s13659-013-0028-5 and is accessible for authorized users.

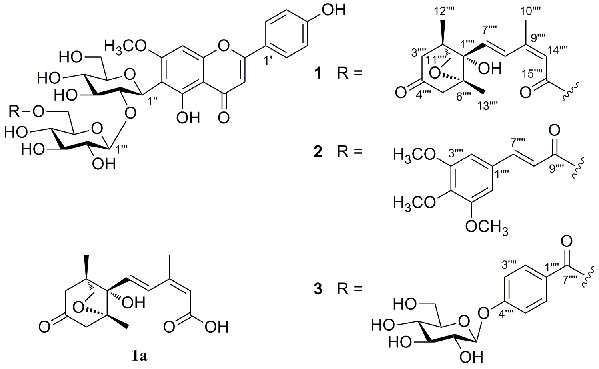

## Introduction

The genus *Ziziphus* (Rhamnaceae), comprises of approximately 170 species and 12 variants, and is distributed in the warm-temperate and subtropical regions throughout the world. Thirteen of which are distributed in the southern and eastern China. Most *Ziziphus* species are important sources for their edible fruits and medicinal uses.[1–3] In the Chinese Pharmacopoeia, the dry seeds of *Z. jujuba* Mill var. *spinosa* (Bunge) Hu ex H. F. Chou, commonly named as Semen Ziziphi Spinosae ("Suan-Zao-Ren"), have been used traditionally to tranquilize and relax the mind, soothing nerves (anxiolytic), and reducing sweating (anti-hydronic) effect. Flavonoids, saponins and alkaloids were previously reported as isolated from the seeds of *Z. jujuba*.[4–6]

On the other hand, *Z. mauritiana* Lam., an evergreen shrub up to 15 m height, is distributed in the southern China. It is also widely growing throughout low-latitudes of Asia, Africa and Australia.[1,2] The dry seeds have been used as a substitute of "Suan-Zao-Ren" (seeds of *Z. jujuba* var. *spinosa*) by local people in Yunnan province of China. Cyclopepeptide,[1,7–12] triterpenes,[1] flavones,[1] steroids,[1,13] and aliphatic compounds[1,14] have previously been identified from the roots, barks and leaves of *Z. mauritiana*. Interestingly, some of these compounds showed antitumor, anti-HIV,[15,16] anti-plasmodial and anti-mycobacterial activities.[12] However, no chemical study was reported on the seeds. As a part of our continuing study of the chief phytotherapeutic components of traditional Chinese medicines (TCM),[17–19] the phytochemical investigation on the seeds of *Z. mauritiana* was carried on. This led to the isolation of 11 spinosin derivatives (**1**–**11**), along with 11 other known compounds (**12**–**22**). Compounds **1**–**3** are new acylated flavonoid *C*-glycosides. The acetylcholinesterase (AChE) inhibitory activities of the major isolates were tested, using tacrine as the positive control.

**Figure 1.** Chemical structures of flavonoids **1**–**3**

## Results and Discussion

The defated MeOH extract of the air-dried seeds of *Z. mauritiana* was applied to column chromatography (CC) over Diaion HP20SS, Sephadex LH-20, Chromatorex ODS, Toyopearl HW40C, silica gel, MCI-gel CHP20P, and RP-18, finally to semi-preparative HPLC, to give three new compounds (**1**–**3**) and 19 known ones. The known compounds (see Electronic Supplementary Material) were determined to

*To whom correspondence should be addressed. E-mail: zhangyj@mail.kib.ac.cn (Y.J. Zhang); xumin@mail.kib.ac.cn (M. Xu)

**Table 1.** $^1$**H (600 MHz) and** $^{13}$**C (150 MHz) NMR spectroscopic data of compound 1 ( DMSO-$d_6$; 313 K; $\delta$ in ppm)**

| pos. | $\delta_H$ ($J$, Hz) | $\delta_C$ | pos. | $\delta_H$ ($J$, Hz) | $\delta_C$ |
|---|---|---|---|---|---|
| 2 | | 164.10/163.90, C | 2''' | 2.84 (t, 8.8) | 74.49/74.43, CH |
| 3 | 6.79/6.72 (s) | 103.12/103.03, CH | 3''' | 3.06 (m) | 76.25/76.19, CH |
| 4 | | 182.23/181.95, C | 4''' | 2.94 (t, 8.9) | 69.12/69.09, CH |
| 5 | | 160.69/159.57, C | 5''' | 2.86 (m) | 73.51/73.44, CH |
| 6 | | 108.89/108.81, C | 6''' | 3.87[a]/3.62 (m) | 62.42/61.65, $CH_2$ |
| 7 | | 165.14/163.67, C | 1'''' | | 81.40/81.37, C |
| 8 | 6.77/6.72 (s) | 90.65/90.10, CH | 2'''' | | 48.66/48.28, C |
| 9 | | 157.03/156.92, C | 3'''' | $H_\beta$ 2.61 (m)/$H_\alpha$ 2.27 (m) | 52.00/51.96, $CH_2$ |
| 10 | | 104.48/104.08, C | 4'''' | | 208.37/208.35, C |
| 1' | | 121.14/121.10, C | 5'''' | $H_\beta$ 2.76 (m)/$H_\alpha$ 2.30 (m) | 53.00, $CH_2$ |
| 2',6' | 7.91/7.91 (d, 8.8) | 128.52/128.49, CH | 6'''' | | 86.04/86.01, C |
| 3',5' | 6.91/6.90 (d, 8.8) | 115.93/115.92, CH | 7'''' | 6.40/6.39 (d, 15.7) | 134.22/134.14, CH |
| 4' | | 161.23, C | 8'''' | 7.89/7.81 (d, 15.7) | 130.35/130.31, CH |
| 7-OMe | 3.89/3.87 (s)[a] | 56.45/56.16, $CH_3$ | 9'''' | | 150.60/150.52, C |
| 1'' | 4.70/4.68 (d,10.1) | 71.07/70.76, CH | 10'''' | 1.91/1.87 (s) | 20.72/20.70, $CH_3$ |
| 2'' | 4.46 (t, 9.4)/4.24[b] | 80.89/79.62, CH | 11'''' | 3.80/3.53 (m) | 76.66, $CH_2$ |
| 3'' | 3.46/3.44[c] | 78.70/78.46, CH | 12'''' | 0.88/0.87 (s) | 15.35/15.28, $CH_3$ |
| 4'' | 3.17[c] | 70.48/70.41, CH | 13'''' | 1.06/1.03 (s) | 19.15/19.11, $CH_3$ |
| 5'' | 3.17[c] | 81.82/81.60, CH | 14'''' | 5.50 (s) | 116.72/116.66, CH |
| 6'' | 3.39[c]/3.69 (m) | 61.55/61.53, $CH_2$ | 15'''' | | 164.95/164.78, C |
| 1''' | 4.25 (d, 7.7)[b] | 105.01/104.88, CH | 5-OH | 13.58/13.46 (s) | |

[a,b]overlapped with each other for the same superscript; [c]overlapped by solvent.

be eight spinosin derivatives [6'''-dihydrophaseoyl- (**4**),[5] 6'''-*p*-coumaroyl- (**5**),[20] 6'''-feruloyl- (**6**),[4] 6'''-sinapoyl- (**7**),[20] 6'''-(4''''-*O*-$\beta$-D-glucopyranosyl)-vanilloyl- (**8**),[6] 6'''-vanilloyl- (**9**)[21] spinosin, spinosin (**10**),[4] and isospinosin (**11**)[4]], two flavone *C*-glycosides [vicenin-2 (**12**),[22] apigenin 6-*C*-$\alpha$-L-rhamnopyranosyl-(1→2)-$\beta$-D-glucopyranoside (**13**),[23]], one triterpenoid saponin [jujuboside A (**14**)[24]], six lignans [pinoresinol-4,4'-di-$\beta$-*O*-D-glucoside (**15**),[25] *erythro*-dihydroxydehydrodiconifery alcohol (**16**),[26] *threo*-dihydroxydehydrodiconifery alcohol (**17**),[26] aegineoside (**18**),[27] (+)-dehydrodi-coniferyl alcohol-4-*O*-$\beta$-D-glucoside (**19**),[28] lariciresinol-4'-*O*-$\beta$-D-glucopyranoside (**20**[29])], one alkaloid [zizyphusine (**21**)[30]] and one amino acid [tryptophan (**22**) [31]], by comparison of their spectroscopic data with the reported literature values.

Compound **1** was isolated as a yellow amorphous powder. Its molecular formula was deduced to be $C_{43}H_{50}O_{19}$ on the basis of HREIMS (*m/z* 870.2999 [M]$^+$). The IR spectrum showed absorption bands at 3430 and 1708 cm$^{-1}$ due to hydroxyl and carbonyl groups, respectively. The UV spectrum exhibited maximum absorptions at 210, 272, and 332 nm. All the protons and carbons of **1** appeared as pair signals in the $^1$H and $^{13}$C NMR spectra, which is characteristic of signals arising from a spinosin skeleton[5]. In addition, the remaining 15 carbon resonances were further classified by DEPT experiment as six quaternary carbons, including a ketone ($\delta_C$ 208.37/208.35, C-4'''') and a carbonyl ($\delta_C$ 164.95/164.78, C-15''''), three olefinic methines, three methylenes including one oxygen-bearing ($\delta_C$ 76.66, C-11'''') and three quaternary methyl ($\delta_C$ 20.72/20.70, 15.35/15.28, 19.15/19.11, C-10'''', C-12'''', C-13''''). The $^1$H NMR spectrum displayed the presence of two *trans*-coupled olefinic protons ($\delta_H$ 6.40/6.39, 7.89/7.81, each 1H, d, $J$ = 15.7 Hz, H-7'''', H-8''''), one singlet olefinic proton at $\delta_H$ 5.50/5.50 (1H, s, H-14'''')] and three methyl singlets [$\delta_H$ 1.91/1.87, 0.88/0.87, 1.06/1.03 (each 3H, s)]. The aforementioned data of **1** were closely related to those of 6'''-dihydrophaseoylspinosin (**4**). The difference between **1** and **4** was the sesquiterpene moiety, featuring with an additional ketone in **1**, relative to the oxygen-bearing methine in **4**. In the HMBC spectrum of **1** (Figure 2), correlations of both H-3'''' ($\delta_H$ 2.61/2.27) and H-5'''' ($\delta_H$ 2.76/2.30) with C-4'''' ($\delta_C$ 208.37/208.35), could assign the additional ketone ($\delta_C$ 208.37/208.35) to be C-4''''. The above data of the sesquiterpene moiety of **1** was essentially identical to those of phaseic acid.[32] The position of the phaseic acid moiety in **1** was revealed to be at C-6''' by the HMBC correlation of the H-6''' ($\delta_H$ 3.87/3.62) with the carbonyl carbon at $\delta_C$ 164.95/164.78 (C-15''''). Other HMBC and $^1$H-$^1$H COSY correlations (Figure 2), with electronic supplementary material further confirmed the structure of **1** as shown in Figure 1.

**Figure 2.** Key HMBC and ROESY correlations of compound **1**

The relative configuration of compound **1** was determined on the basis of the coupling constants and ROESY experiment (Figure 2). The *trans* double bond between C-7'''' and C-8'''' was deduced due to the large coupling constant (15.7 Hz) of $J_{7'''',8''''}$, while the *cis* double bond between C-9'''' and C-14'''' was indicated by the ROESY correlations of the olefinic H-14'''' ($\delta_H$ 5.50/5.50) with Me-10'''' ($\delta_H$ 1.91/1.87). In the ROESY spectrum of **1**, correlations of H-7'''' and H-8'''' with both Me-12'''' and Me-13'''' were observed. In addition, the H-3$\alpha$'''' and H-3$\beta$'''' were correlated with H-11'''' and Me-12'''', respectively. These data revealed that the unsaturated side chain -CH(7)=CH(8)-C(9)=CH(14)-COO(15)- of phaseic acid moiety in **1** oriented to the same side of both Me-12'''' and Me-13'''', while the $CH_2$-11'''' and O-C(6'''') were oriented to the opposite side of Me-12'''', Me-13'''' and C-7'''' side chain. Alkaline hydrolysis of **1** with 0.5% NaOH yielded spinosin (**10**) and phaseic acid (**1a**). Compound **1a** showed the similar NMR spectroscopic data and optical rotation value ($[\alpha]_D^{20}$ –

**Table 2.** **$^1$H (600 MHz) and $^{13}$C (150 MHz) NMR spectroscopic data of compounds 2 and 3 ( DMSO-$d_6$; 313 K; $\delta$ in ppm)**

| | 2 | | 3 | |
|---|---|---|---|---|
| position | $\delta_H$ (*J*, Hz) | $\delta_C$ | $\delta_H$ (*J*, Hz) | $\delta_C$ |
| 2 | | 164.10/163.89, C | | 163.89/163.84, C |
| 3 | 6.69/6.51 (s) | 103.17/102.92, CH | 6.70/6.46 (1H, s) | 102.89/102.74, CH |
| 4 | | 182.22/181.78, C | | 182.23/181.69, C |
| 5 | | 160.81/159.46, C | | 160.70/159.48, C |
| 6 | | 108.76/108.74, C | | 108.81/108.66, C |
| 7 | | 165.23/163.60, C | | 165.09/163.56, C |
| 8 | 6.69/6.69 (s) | 90.55/89.88, CH | 6.64/6.53 (1H, s) | 90.47/89.85, CH |
| 9 | | 157.01/156.85, C | | 156.91/156.73, C |
| 10 | | 104.45/103.96, C | | 104.41/103.90, C |
| 1' | | 121.13/121.09, C | | 121.02/120.86, C |
| 2',6' | 7.80/7.79 (d, 8.8) | 128.47/128.39, CH | 7.82/7.75 (d, 8.7) | 128.46/128.41, CH |
| 3',5' | 6.88/6.83 (d, 8.8) | 115.76/115.70, CH | 6.93/6.92 (d, 8.7) | 115.77/115.66, CH |
| 4' | | 161.23/161.15, C | | 161.39/161.34, C |
| 7-$OCH_3$ | 3.90/3.87 (s) | 56.40/ 56.10, $CH_3$ | 3.87/3.80 (s) | 56.37/56.09, $CH_3$ |
| 1'' | 4.70/4.69 (d, 9.8) | 70.98/ 70.64, CH | 4.69/4.68 (d, 9.7) | 71.02/70.67, CH |
| 2'' | 4.49 (t, 9.3)/4.25 (t, 9.3) | 81.65/ 80.13, CH | 4.45 (t, 9.4)/4.23 (t, 9.4) | 81.59/80.26, CH |
| 3'' | 3.44 (m) | 78.81/78.63, CH | 3.43[a] | 78.49, CH |
| 4'' | 3.16[a] | 70.33/70.27, CH | 3.16[a] | 70.33/70.27, CH |
| 5'' | 3.16[a] | 81.93, CH | 3.16[a] | 81.91/81.90, CH |
| 6'' | 3.82[b]/3.38 (m) | 61.53/61.49, $CH_2$ | 3.70 (1H, m)/3.37[a] | 61.52/61.48, $CH_2$ |
| 1''' | 4.31/4.30 (d, 7.3) | 105.76/105.70, CH | 4.28/4.27 (d, 7.8) | 105.61/105.16, CH |
| 2''' | 2.93 (t, 8.3)/2.90 (t, 8.3) | 74.41/74.38, CH | 2.94 (t, 8.1)/2.89 (t, 8.1) | 74.54/74.51, CH |
| 3''' | 3.11 (m) | 76.31, CH | 3.11[c] | 76.15/76.11, CH |
| 4''' | 3.09 (m) | 68.86/68.51, CH | 3.08[c] | 68.96/68.84, CH |
| 5''' | 2.98 (m) | 73.25/72.94, CH | 2.97 (1H, m) | 73.15, CH |
| 6''' | 4.05/3.65 (m) | 62.41/62.29, $CH_2$ | 4.04 (1H, m)/3.61 (1H, m) | 62.74/62.21, $CH_2$ |
| 1'''' | | 129.46/129.21, C | | 122.93/122.80, C |
| 2'''' | 6.86/6.69 (s) | 105.53/105.18, CH | 7.61/7.49 (d, 8.7) | 130.83/130.64, CH |
| 3'''' | | 152.98/152.87, C | 7.04/6.95 (d, 8.8) | 115.88/115.86, CH |
| 4'''' | | 139.43/139.38, C | | 161.00/160.95, C |
| 5'''' | | 152.98/152.87, C | 7.04/6.95 (d, 8.8) | 115.88/115.86, CH |
| 6'''' | 6.86/6.69 (s) | 105.53/105.18, CH | 7.61/7.49 (d, 8.7) | 130.83/130.64, CH |
| 7'''' | 7.22/7.06 (d, 15.8) | 144.32/144.15, CH | | 164.74/164.65, C |
| 8'''' | 6.40/6.30 (d, 15.8) | 116.80/116.29, CH | | |
| 9'''' | | 166.08/165.96, C | | |
| 3''''-$OCH_3$ | 3.82/3.79 (s)[b] | 55.98/55.90, $CH_3$ | | |
| 4''''-$OCH_3$ | 3.71/3.70 (s) | 60.08/60.06, $CH_3$ | | |
| 5''''-$OCH_3$ | 3.82/3.79 (s)[b] | 55.98/55.90, $CH_3$ | | |
| 1''''' | | | 5.00/4.96 (d, 7.5) | 100.03/ 99.91, CH |
| 2''''' | | | 3.28[a] | 73.18, CH |
| 3''''' | | | 3.34[a] | 77.15/76.59, CH |
| 4''''' | | | 3.18[a] | 69.71/69.68, CH |
| 5''''' | | | 3.44[a] | 78.77/78.79, CH |
| 6''''' | | | 3.70 (1H, m)/3.50[a] | 60.69/60.67, $CH_2$ |
| 5-OH | 13.59 (s) | | 13.64 (s) | |

[a]overlapped by solvent; [b]overlapped with each other.

21.7, *c* 0.098, MeOH) with those of the reported (–)-phaseic acid ($[\alpha]_D^{16}$ – 18.0, *c* 0.10, MeOH).[32,33] Therefore, the structure of compound **1** was deduced as shown in Figure 1 and named 6'''-(–)-phaseoylspinosin.

Compound **2**, a yellow amorphous powder, has a molecular formula determined to be $C_{40}H_{44}O_{19}$, as deduced from the HREIMS (*m/z* 828.2475, calcd. for 828.2477). The $^1$H and $^{13}$C NMR spectra of **2** exhibited the characteristic features of a spinosin skeleton. In addition, a set of signals arising from one carbonyl ($\delta_C$ 166.08/165.96, C-9''''), three methoxyl [$\delta_C$ 55.98/55.90 ($OCH_3$ × 2), 60.08/60.06), four olefinic methines [$\delta_C$ 105.53/105.18 (CH × 2), 144.32/144.15 (CH), 116.80/116.29 (CH)], and four aromatic quaternary carbons [$\delta_C$ 129.46/129.21 (C), 152.98/152.87 (C × 2), 139.43/139.38 (C)] were observed in the $^{13}$C NMR (DEPT) spectra. The $^1$H NMR spectrum displayed the existence of a symmetric 1,3,4,5-tetra-substituted aromatic ring [$\delta_H$ 6.86/6.69 (2H, s)] , one *trans* double bond [$\delta_H$ 7.22/7.06, 6.40/6.30 (each 1H, d, *J* = 15.8 Hz, H-7'''', H-8'''')] and three methoxyl groups [$\delta_H$ 3.82/3.79 (6H, s, 3'''', 5''''-OMe); $\delta_H$ 3.71/3.70 (3H, s, 4''''-OMe)]. The aforementioned data suggested that **2** was a spinosin derivative acylated with a 3,4,5-trimethoxy-(*E*)-cinnamoyl moiety.[34] In the HMBC spectrum of **2** (Figure 3), correlations between the H-6''' ($\delta_H$ 4.05/3.65) and C-9'''' ($\delta_C$ 166.08/165.96) indicated the linkage of the cinnamoyl moiety with C-6''' ($\delta_C$ 62.41/62.29) of spinosin unit. Other HMBC correlations (Figure 3) were used to further confirm the structure of **2** as shown in Figure 1. Thus, the structure of compound **2** was determined as 6'''-(3'''',4'''',5''''-trimethoxy)-(*E*)-cinnamoylspinosin.

Compound **3** was isolated as a yellow amorphous powder. On the basis of the HREIMS (*m/z* 890.2523, calcd. for

**Figure 3.** Key HMBC (H→C) correlations of compound **2**

890.2481), its molecular formula was determined to be $C_{41}H_{46}O_{22}$. The $^1$H and $^{13}$C NMR spectra also showed the presence of a spinosin skeleton in **3**. In addition, an AA'BB' coupled aromatic system [$\delta_H$ 7.61/7.49 (2H, d, $J$ = 8.7 Hz, H-2'''', 6'''') and $\delta_H$ 7.04/6.95 (2H, d, $J$ = 8.8 Hz, H-3'''', 5'''')] was observed in the $^1$H NMR spectrum, together with an additional anomeric proton at $\delta_H$ 5.00/4.96 (1H, d, $J$ = 7.5 Hz). The $^{13}$C NMR (DEPT) spectra showed the occurance of a carboxyl ($\delta_C$ 164.74/164.65, C-7'''') and six aromatic carbons arising from a benzoyl unit, and six oxygen-bearing alphatic carbons due to a glucosyl moiety. The above data revealed that compound **3** was an analogue of **8**,[6] except for the absence of one methoxyl group associated with the benzoyl moiety, related to **8**. The HMBC correlations of the anomeric proton at H-1''''' ($\delta_H$ 5.00/4.96) with C-4'''' ($\delta_C$ 161.00/160.95), H-2'''',6'''' ($\delta_H$ 7.61/7.49) with C-4'''' ($\delta_C$ 161.00/160.95) and C-7'''' (164.74/164.65), and H-3'''',5'''' ($\delta_H$ 7.04/6.95) with C-1'''' ($\delta_C$ 122.93/122.80) suggested the additional glucosyl unit was connected to the benzoyl C-4'''' position. The linkage of the benzoyl group with C-6''' of spinosin moiety were supported by the HMBC correlations from H-6''' ($\delta_H$ 4.04/3.61) to C-7'''' ($\delta_C$ 164.74/164.65). Consequently, the structure of compound **3** was determined to be 6'''-(4''''-*O*-*β*-D-glucopyranosyl)-benzoylspinosin.

It is note that the $^1$H and $^{13}$C NMR data of the spinosin (**10**) and its derivatives **1**–**9** exhibited doubling of signals at room temperature due to the rotational isomers produced by the rotational barriers 7-$OCH_3$ in flavones-6-*C*-glycoside.[4]

As compared with the positive control, tacrine (59.0%), compounds **1**, **2**, **4**, **7** and **14** displayed moderate AChE inhibitory activity with inhibition values of 24.9%, 33.9%, 26.9%, 28.8% and 46.2%, respectively, at a concentration of 1 $\mu$M, by the spectrophotometric method developed by Ellman et al.[35] with slight modification. Interstingly, AChE inhibition of compound **8** was only 7.8% at 1 $\mu$M concentration.

## Experimental Section

**General Experimental Procedures.** Optical rotations were determined with a Jasco P-1020 polarimeter. UV (in MeOH) spectra were obtained with the Shimadzu UV-2401 PC spectraphotometer. The Bruker Tensor-27 infrared spectrophotometer was used for IR spectra as KBr pellets. ESIMS and HREIMS spectra were recorded on API QSTAR Pulsar spectrometer and Waters Autospec Premier P776, respectively. 1D and 2D NMR spectra were performed on Bruker Avance III-600MHz spectrometers with TMS as an internal standard. Semi-preparative and preparative HPLC studies were performed on an Agilent 1260 and Gilson GX-27X liquid chromatograph. TLC was performed on precoated TLC plates (0.2–0.25 mm thickness, GF254 Si gel 60, Qingdao Marine Chemical Co., Ltd.) with compounds visualized by spraying the dried plates with 10% aqueous $H_2SO_4$ followed by heating until the plate was dry. Silical gel (200–300 mesh, Qingdao Marine Chemical Co., Ltd.), Diaion HP20SS (Mitsubishi Chemical Co., Ltd.), Lichroprep RP-18 (40–63 $\mu$m, Merck), Sephadex LH-20 (25–100 $\mu$m, Pharmacia Fine Chemical Co., Ltd.), Chromatorex ODS (100–200 mesh, Fuji Silysia Chemical Co., Ltd.), Toyopearl HW40C (50–100 $\mu$m, Tosoh Co., Ltd.) and MCI-gel CHP20P (75–150 $\mu$m, Mitsubishi Chemical Co., Ltd.) were used for column chromatography (CC). The chemicals used for bioassay including *S*-acetylthiocholine iodide, *S*-butyrylthiocholine iodide, 5,5'-dithio-bis-(2-nitro-benzoic) acid (DTNB), and AChE was derived from human erythrocytes purchased from Sigma Chemicals.

**Plant Material.** The seeds of *Z. mauritiana* were collected from the herbal medicine market in Kunming, Yunnan Province, China, in July, 2011, and identified by Prof. Chong-Ren Yang (Kunming Institute of Botany, Chinese Academy of Sciences). A voucher sample (KIB-Z-00331) has been deposited in the State Key Laboratory of Phytochemistry and Plant Resources in West China, Kunming Institute of Botany, Chinese Academy of Sciences.

**Extraction and Isolation.** The air-dried seeds (20 kg) of *Z. mauritiana* were powdered, and extracted three times per 2 hours with MeOH at 60 °C. The extracts was suspended in $H_2O$ and degreased by petroleum ether. The $H_2O$-soluble layer (1.2 kg) was applied to a Diaion HP20SS column, eluting with $H_2O$/MeOH (1:0→0:1), to give fractions I–IV. Compound **10** (13.6 g) was obtained by crystallization from fraction III, and the remaining part was further subjected to Sephadex LH-20 chromatography column, eluting with MeOH-$H_2O$ (10:90, 30:70, 50:50, 70:30 and 100:0) to derive the subfractions (III-1 and III-2). Subfraction III-1 (20.5 g) was subjected to Chromatorex ODS column, and eluted with $H_2O$/MeOH (1:0 →0:1), to derive seven fractions (III-1-1–7). Fraction III-1-3 (2.0 g) was subjected to Sephadex LH-20, MCI-gel, RP-18, silica gel and semi-preparative HPLC, and obtained compounds **12** (7 mg), **13** (8 mg), **16** (4 mg), **17** (5 mg), **18** (31 mg) and **22** (26 mg). Fraction III-1-4 (5.0 g) was successively separated by Toyopearl HW40C, Sephadex LH-20, silica gel and Sephadex LH-20, then compound **3** (40 mg), **11** (40 mg), **19** (69 mg) and **20** (63 mg) were obtained. Compound **8** (10 mg) was obtained from Fr. III-1-6 (1.1 g) by repeated CC over Toyopearl HW40C, MCI, RP-18 and finally purified by HPLC using MeCN-$H_2O$ (18:82) as elution. The sub-fraction III-1-7 (3.2 g) was successively subjected to Toyopearl HW40C, MCI-gel, Sephadex LH-20, and silica gel to afford compounds **1** (139 mg), **4** (170 mg), **7** (113 mg) and **15** (47 mg). Furthermore, fraction III-2 (12.6 g) was successively separated by Rp-18, Toyopearl HW40C and preparative HPLC using MeCN-$H_2O$, and purified by semi-preparative HPLC using MeCN-$H_2O$, and isolate compound **9** (4 mg). Similarly, Fraction IV was subjected to Sephadex LH-20 chromatography column, and eluted with MeOH-$H_2O$ (30:70, 50:50, 70:30 and 90:10) and was successively separated by Toyopearl HW40C, Rp-18, silica gel, Sephadex LH-20, MCI-gel and silica gel were used to isolate compounds **2** (280 mg), **5** (11 mg), **6** (200 mg), **14** (137 mg) and **21** (109 mg).

**6'''-(–)-Phaseoylspinosin (1):** yellow amorphous powder; $[\alpha]_D^{20}$ – 31.7 (*c* 0.2, MeOH); UV (MeOH) $\lambda_{max}$ (log $\varepsilon$) 332 (4.31), 272 (4.59) and 210 (4.56) nm; IR (KBr) $\nu_{max}$ 3430, 2928, 2883, 1708, 1653, 1607, 1511, 1491, 1448, 839 and 779 $cm^{-1}$, $^1$H and $^{13}$C NMR data see Table 1; ESIMS *m/z* 893 [M + Na]$^+$; HREIMS *m/z* 870.2999 (calcd. for $C_{43}H_{50}O_{19}$ [M]$^+$, 870.2946).

**6'''-(3'''',4'''',5''''-Trimethoxy)-cinnamoylspinosin (2):** yellow amorphous powder; $[\alpha]_D^{19}$ – 63.4 (*c* 0.2, MeOH); UV (MeOH) $\lambda_{max}$ (log $\varepsilon$) 309 (4.52) and 206 (4.65) nm; IR (KBr) $\nu_{max}$ 3411, 2938, 1706, 1654, 1607, 1508, 1491, 1450, 836 and 778 $cm^{-1}$, $^1H$ and $^{13}C$ NMR data see Table 2; ESIMS *m/z* 851 $[M + Na]^+$; HREIMS *m/z* 828.2475 (calcd. for $C_{40}H_{44}O_{19}$ $[M]^+$, 828.2477).

**6'''-(4''''-*O*-*β*-D-Glucopyranosyl)-benzoylspinosin (3):** yellow amorphous powder; $[\alpha]_D^{19}$ – 83.2 (*c* 0.2, MeOH); UV (MeOH) $\lambda_{max}$ (log $\varepsilon$) 333 (4.31), 307 (4.25), 251 (4.42) and 204 (4.68) nm; IR (KBr) $\nu_{max}$ 3423, 2919, 1704, 1653, 1608, 1510, 1491, 1449, 839 and 770 $cm^{-1}$, $^1H$ and $^{13}C$ NMR data see Table 2; ESIMS *m/z* 913 $[M + Na]^+$ ; HREIMS *m/z* 890.2523 (calcd. for $C_{41}H_{46}O_{22}$ $[M]^+$, 890.2481).

**Alkaline Hydrolysis of 1–3.** A solution of compound **1** (50 mg) in 0.5% NaOH (2 mL) was stirred at 65 °C for 1 h. The reaction mixture was partitioned with $H_2O$ and *n*-BuOH, successively. Further CC over Sephadex LH-20 to afford spinosin (20 mg) from the *n*-BuOH layer. The water layer was neutralized with 0.5 N HCl solution and extracted with $CHCl_3$. The organic layer was subjected to preparative TLC over silica gel ($CHCl_3$-MeOH, 20:1) to afford (–)-phaseic acid (**1a**, 1.9 mg): white amorphous powder; $[\alpha]_D^{20}$ – 21.7 (*c* 0.098, MeOH); ESIMS *m/z* 303 $[M + Na]^+$; $^1H$ NMR (600 MHz, DMSO-$d_6$) : $\delta_H$ 0.87 (3H, s, H-12), 1.07 (3H, s, H-13), 1.97 (3H, s, H-10), 2.29 (1H, d, *J* = 17.6 Hz, H-3a), 2.50 (1H, overlapped with solvent, H-5a), 2.67 (1H, d, *J* = 17.6 Hz, H-3b), 2.78 (1H, d, *J* = 17.4 Hz, H-5b), 3.51 (1H, d, *J* = 7.4 Hz, H-11a), 3.75 (1H, d, 7.4 Hz, H-11b), 5.70 (1H, s, H-14), 6.35 (1H, d, *J* = 15.6 Hz, H-7), 8.02 (1H, s, *J* = 15.6 Hz, H-8). $^{13}C$ NMR (150 MHz, DMSO-$d_6$): $\delta_C$ 15.49 (C-12), 19.30 (C-13), 20.91 (C-10), 48.31 (C-2), 52.04 (C-3), 53.13 (C-5), 76.71 (C-11), 81.35 (C-1), 86.14 (C-6), 121.34 (C-14), 131.14 (C-8), 132.26 (C-7), 150.8 (C-9), 168.3 (C-15), 208.71 (C-4)]. TLC analysis also indicated the presence of spinosin in the aqueous layer ($CHCl_3$-MeOH-$H_2O$ 8:2:0.2, $R_f$ 0.23). Similarly to **1**, alkaline hydrolysis of **2** and **3** (each 10 mg) was carried out, separatedly. The existence of spinosin was confirmed by TLC analysis ($CHCl_3$-MeOH-$H_2O$ 8:2:0.2, $R_f$ 0.23).

**Acid Hydrolysis of 1–3.** Compounds **1–3** (each 10 mg) was dissolved in 2 N HCl (2 mL) and refluxed at 80 °C for 10 h. The reaction mixture was neutralized with Amberlit IRA-401 and a sugar residue was obtained. Identification of D-glucose was performed by comparison of OD (+) with authentic samples.

**Acetylcholinesterase (AChE) Inhibitory Activity.** The AChE inhibitory activity was assayed using the spectrophotometric method developed by Ellman et al.[34] with slightly modification. In brief, the reaction mixture (200 $\mu$L in total) containing phosphate buffer (pH 8.0), testing compound (50 $\mu$M in DMSO), and AChE (0.02 U/mL), was incubated for 20 min (30 °C). Then, the reaction was initiated by the addition of 40 $\mu$L of solution containing DTNB (0.625 mM) and acetylthiocholine iodide (0.625 mM). The hydrolysis of acetylthiocholine was monitored at 405 nm every 30 seconds for one hour. Tacrine was used as positive control with final concentration of 0.333 $\mu$M. All the reactions were performed in triplicate. The percentage inhibition was calculated as follows:

$$\% \text{ inhibition} = (E - S) / E \times 100$$

(E is the activity of the enzyme without test compound and S is the activity of enzyme with the test compound).

## Electronic Supplementary Material

Supplementary material is available in the online version of this article at http://dx.doi.org/10.1007/s13659-013-0028-5 and is accessible for authorized users.

## Acknowledgments

We are grateful to the members of the analytical group of our institute for the measurement of spectroscopic data. The authors are sincerely grateful to Prof. Huai-Rong Luo for the AChE inhibitory activity bioassay. This work was supported by the 973 Program of Science and Technology of China (2011CB915503) and the Fourteenth Candidates of the Young Academic Leaders of Yunnan Province (Min Xu, 2011CI044).

**Open Access** This article is distributed under the terms of the Creative Commons Attribution License which permits any use, distribution, and reproduction in any medium, provided the original author(s) and source are credited.

## Electronic supplementary material

Supplementary material, approximately 846 KB.
