# Supplementary material for: New spinosin derivatives from the seeds of Ziziphus mauritiana
Source: Nat Prod Bioprospect. 2013 May 7;3(3):93–8. doi: 10.1007/s13659-013-0028-5 (PMC4131668; doi:10.1007/s13659-013-0028-5)

Electronic Supplementary Material

New spinosin derivatives from the seeds of *Ziziphus mauritiana*

Bin WANG,[a,b] Hong-Tao ZHU,[a] Dong WANG,[a] Chong-Ren YANG,[a] Min XU,[a,]* and Ying-Jun ZHANG[a,]*

[a]State Key Laboratory of Phytochemistry and Plant Resources in West China, Kunming Institute of Botany, Chinese Academy of Sciences, Kunming 650201, China

[b]University of Chinese Academy of Sciences, Beijing 100049, China

Received 28 March 2013; Accepted 16 April 2013

© The Author(s) 2013. This article is published with open access at Springerlink.com

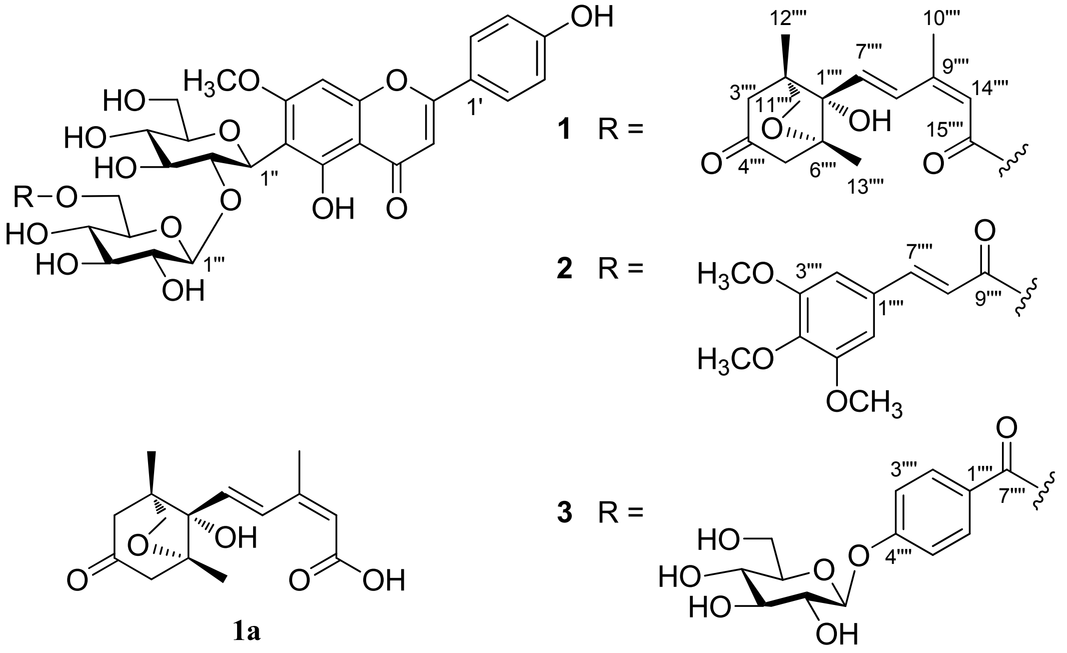

Structures of compounds **1**–**3**

*To whom correspondence should be addressed. E-mail: zhangyj@mail.kib.ac.cn (Y.J. Zhang); xumin@mail.kib.ac.cn (M. Xu)

Fig. 1. ^{1}H NMR (600 MHz, 313 K) spectrum of compound **1** in DMSO- d_6.
Fig. 2. ^{13}C NMR (150 MHz, 313 K) spectrum of compound **1** in DMSO- d_6.
Fig. 3. HSQC (313 K) spectrum of compound **1** in DMSO- d_6.
Fig. 4. HMBC (313 K) spectrum of compound **1** in DMSO-d_6.
Fig. 5. ROESY (313 K) spectrum of compound **1** in DMSO-d_6.
Fig. 6. ^{1}H-^{1}H COSY (313 K) spectrum of compound **1** in DMSO-d_6.
Fig. 7. HREIMS of compound **1**.
Fig. 8. ^{1}H NMR (600 MHz, 313 K) spectrum of compound **2** in DMSO- d_6.
Fig. 9. ^{13}C NMR (150 MHz, 313 K) spectrum of compound **2** in DMSO- d_6.
Fig. 10. HSQC (313 K) spectrum of compound **2** in DMSO- d_6.
Fig. 11. HMBC (313 K) spectrum of compound **2** in DMSO-d_6.
Fig. 12. HREIMS of compound **2**.
Fig. 13. ^{1}H NMR (600 MHz, 313 K) spectrum of compound **3** in DMSO- d_6.
Fig. 14. ^{13}C NMR (150 MHz, 313 K) spectrum of compound **3** in DMSO- d_6.
Fig. 15. HSQC (313 K) spectrum of compound **3** in DMSO- d_6.
Fig. 16. HMBC (313 K) spectrum of compound **3** in DMSO-d_6.
Fig. 17. HREIMS of compound **3**.
Fig. 18. Chemical structures of known compounds **4**–**22**.

Fig. 1. ^{1}H NMR (600 MHz, 313 K) spectrum of compound **1** in DMSO- d_6.

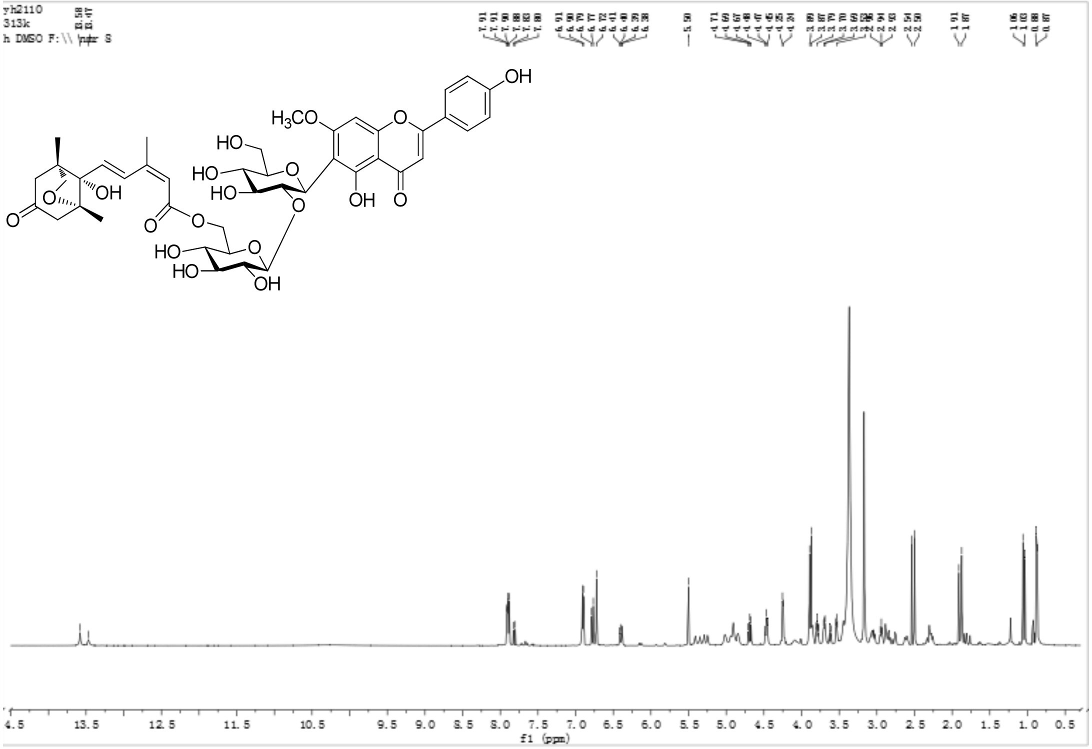

Fig. 2. ^{13}C NMR (150 MHz, 313 K) spectrum of compound **1** in DMSO- d_6.

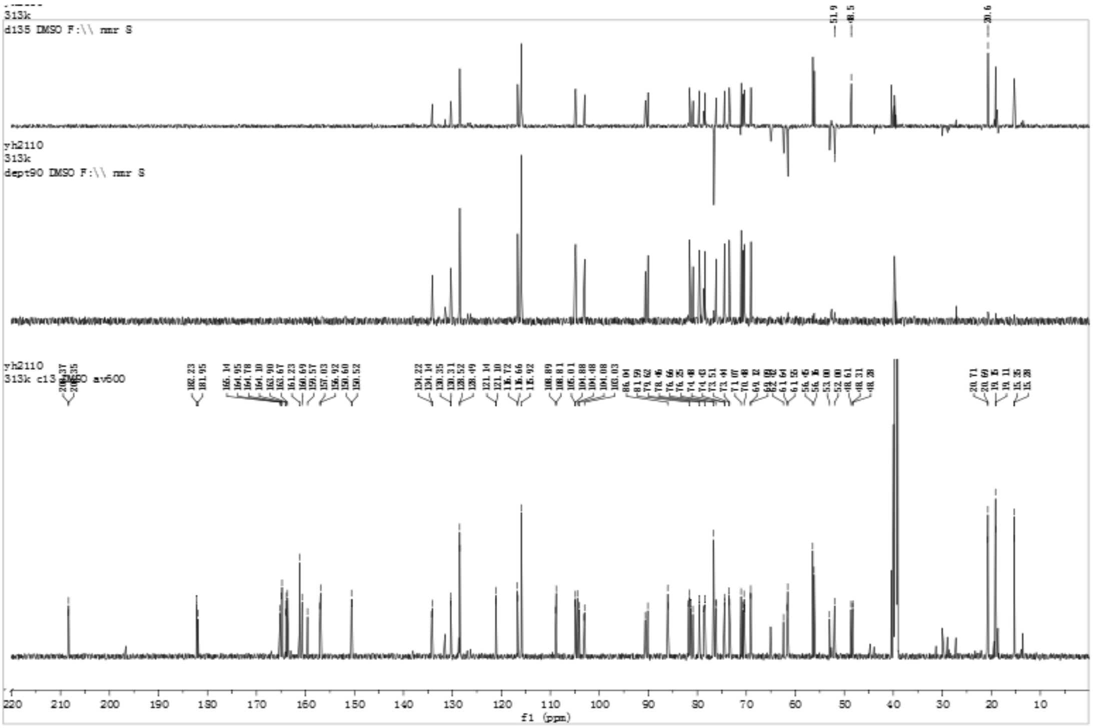

Fig. 3. HSQC (313 K) spectrum of compound **1** in DMSO- d_6.

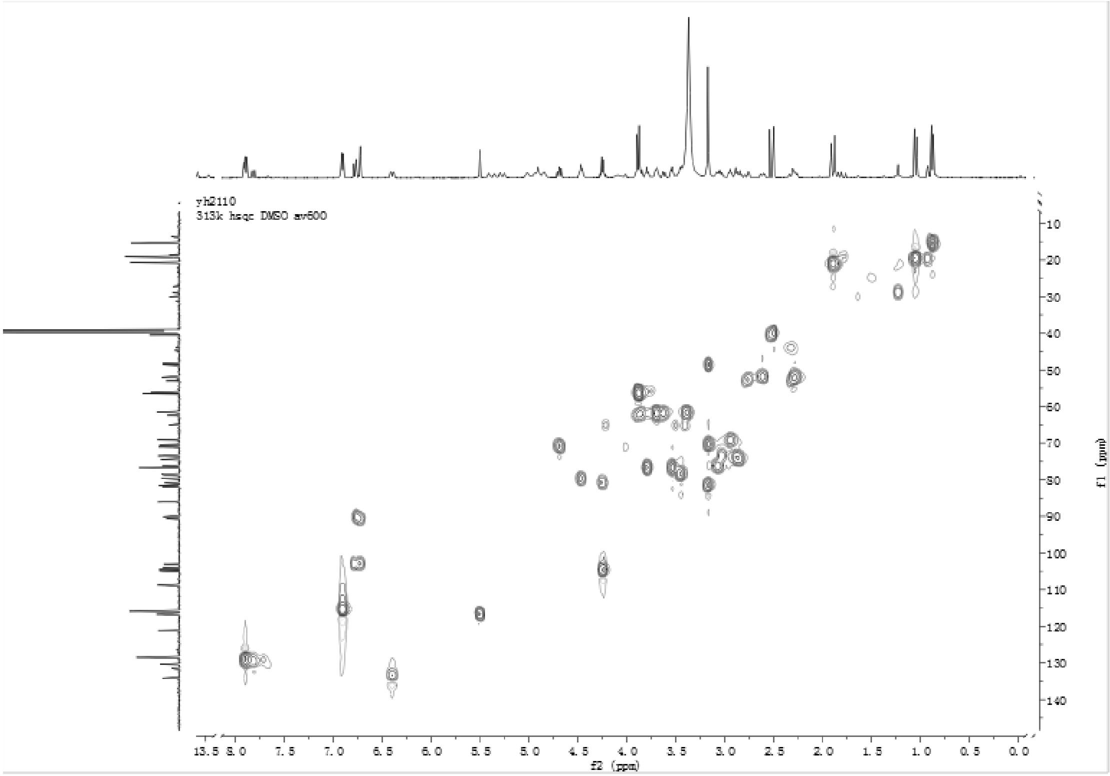

Fig. 4. HMBC (313 K) spectrum of compound **1** in DMSO-d_6.

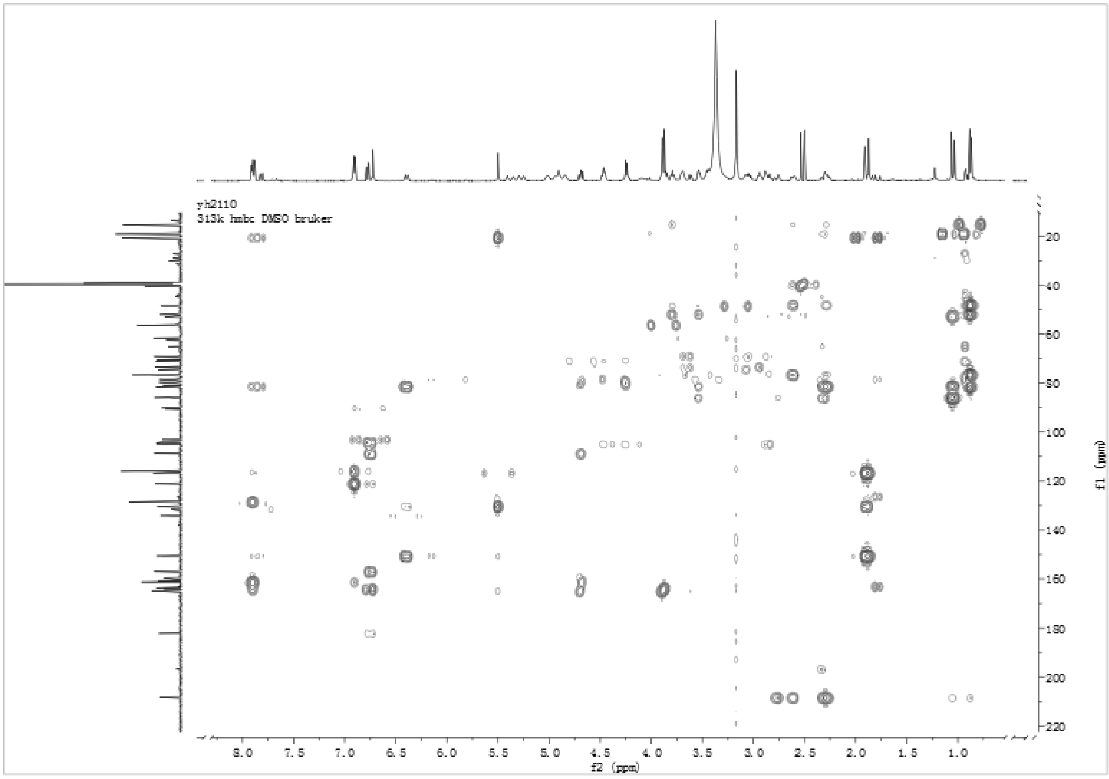

Fig. 5. ROESY (313 K) spectrum of compound **1** in DMSO-d_6.

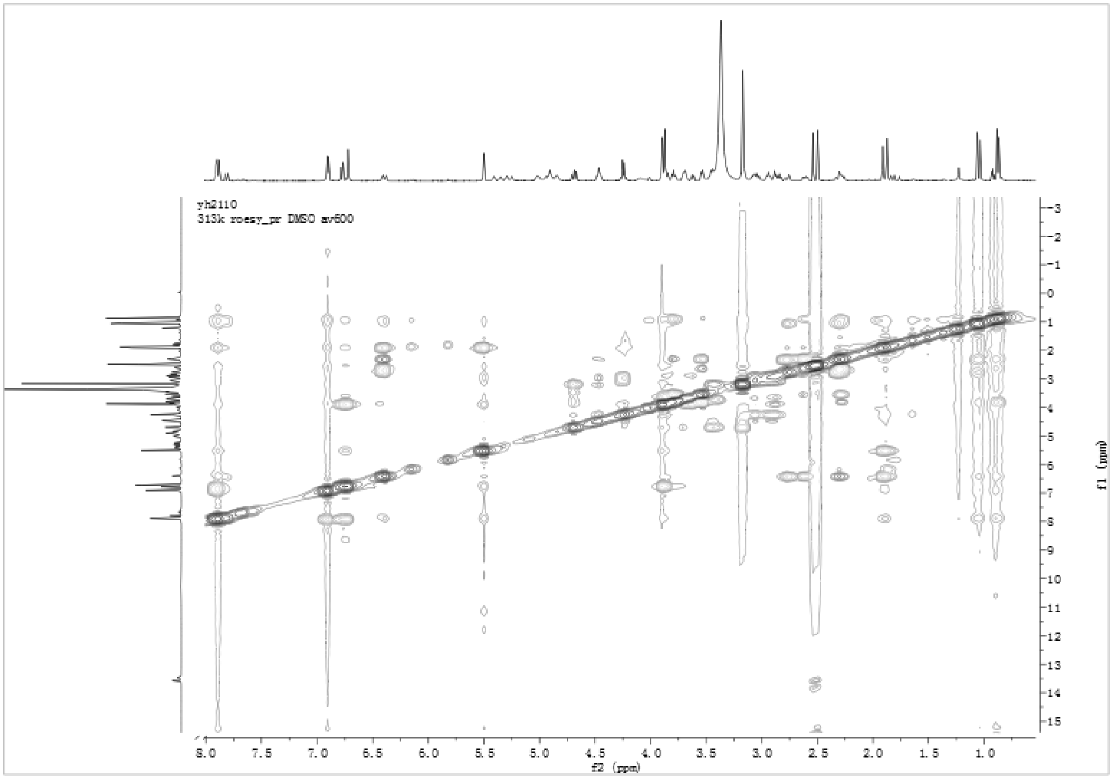

Fig. 6. ^{1}H-^{1}H COSY (313 K) spectrum of compound **1** in DMSO-d_6.

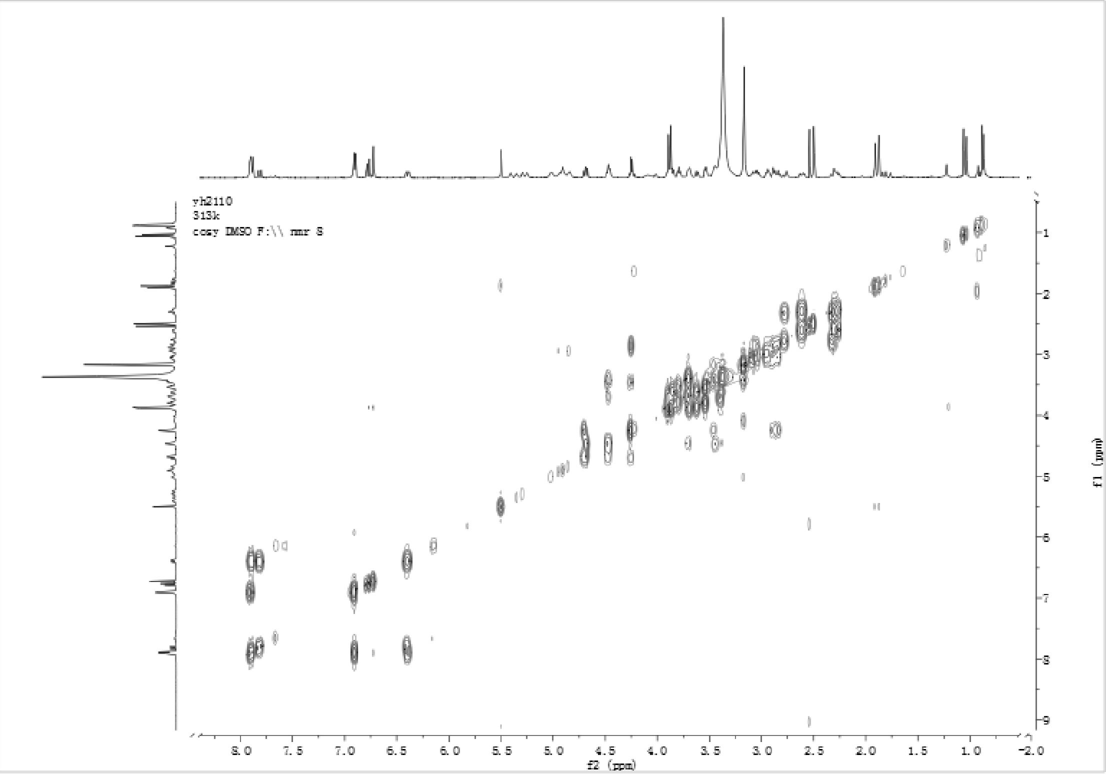

Fig. 7. HREIMS of compound **1**.

Elemental Composition Report **Page 1**

Single Mass Analysis

Tolerance = 10.0 PPM / DBE: min = -10.0, max = 120.0

Selected filters: None

Monoisotopic Mass, Odd and Even Electron Ions

26 formula(e) evaluated with 1 results within limits (up to 51 closest results for each mass)

Elements Used:

C: 0-200 H: 0-400 O: 18-20

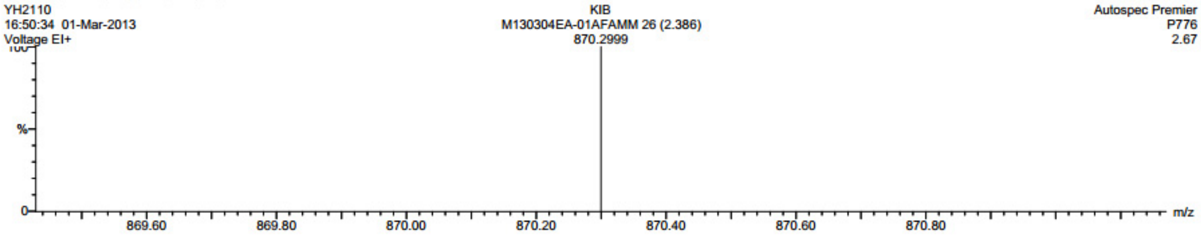

Minimum:				-10.0		
Maximum:		200.0	10.0	120.0		
Mass	Calc. Mass	mDa	PPM	DBE	i-FIT	Formula
870.2999	870.2946	5.3	6.1	19.0	5546025.0	C43 H50 O19

Fig. 8. ^{1}H NMR (600 MHz, 313 K) spectrum of compound **2** in DMSO- d_6.

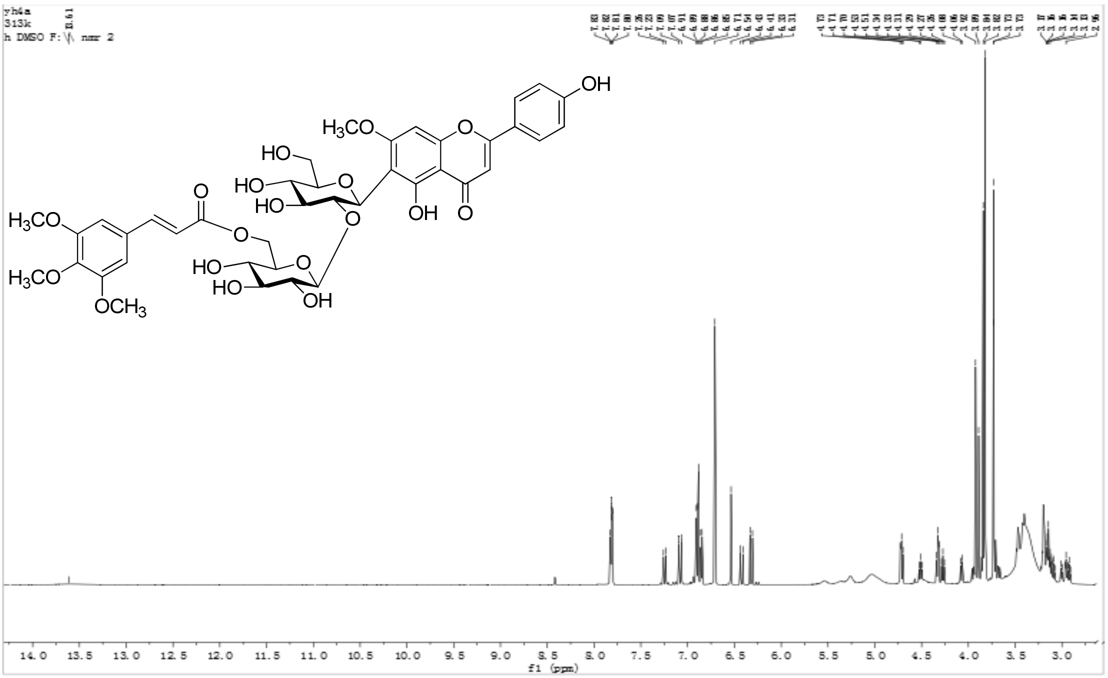

Fig. 9. ^{13}C NMR (150 MHz, 313 K) spectrum of compound **2** in DMSO- d_6.

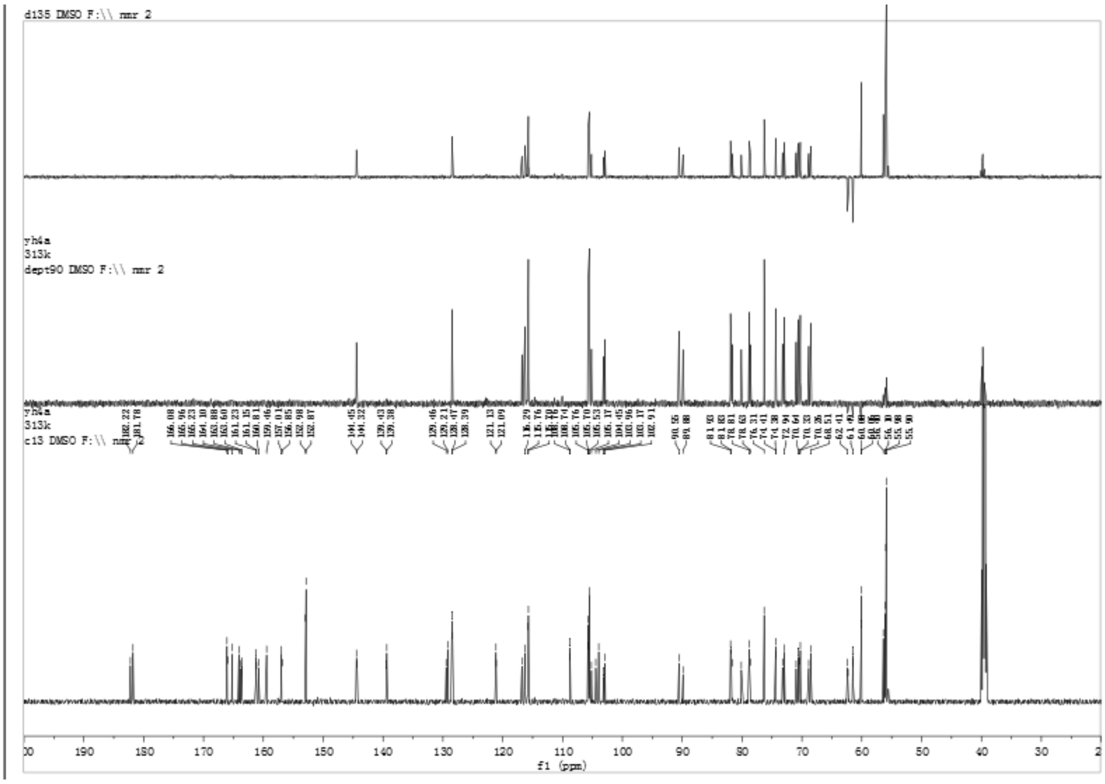

Fig. 10. HSQC (313 K) spectrum of compound **2** in DMSO- d_6.

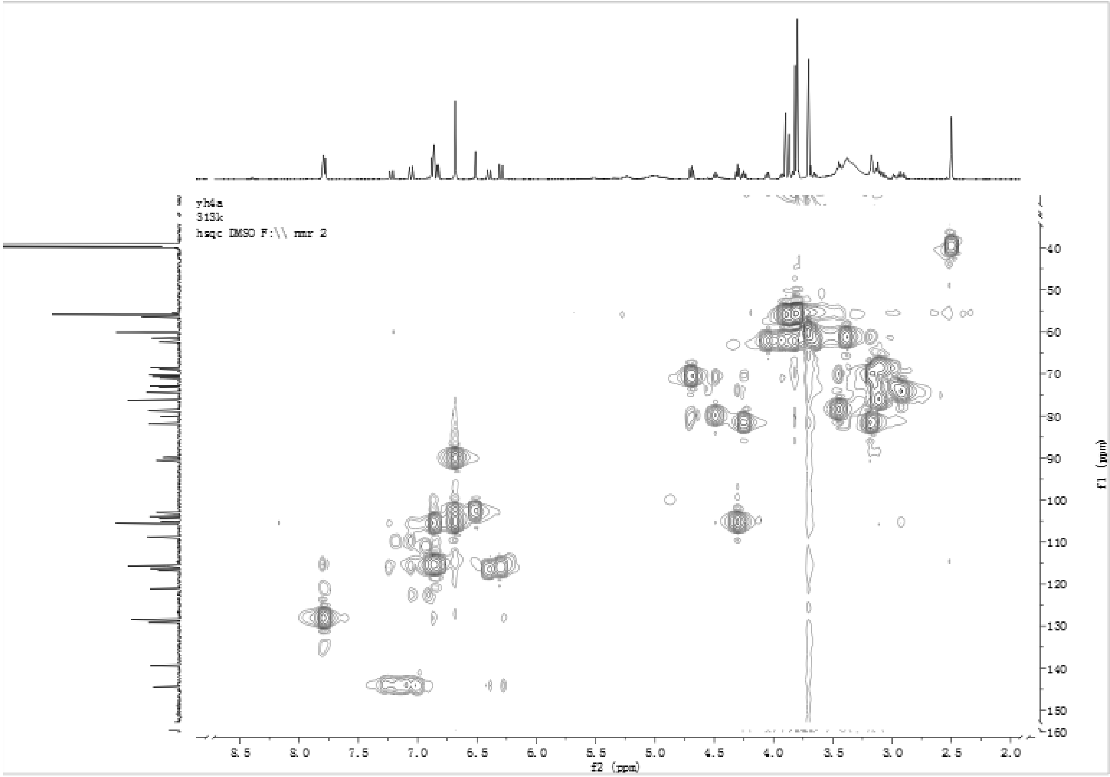

Fig. 11. HMBC (313 K) spectrum of compound **2** in DMSO-d_6.

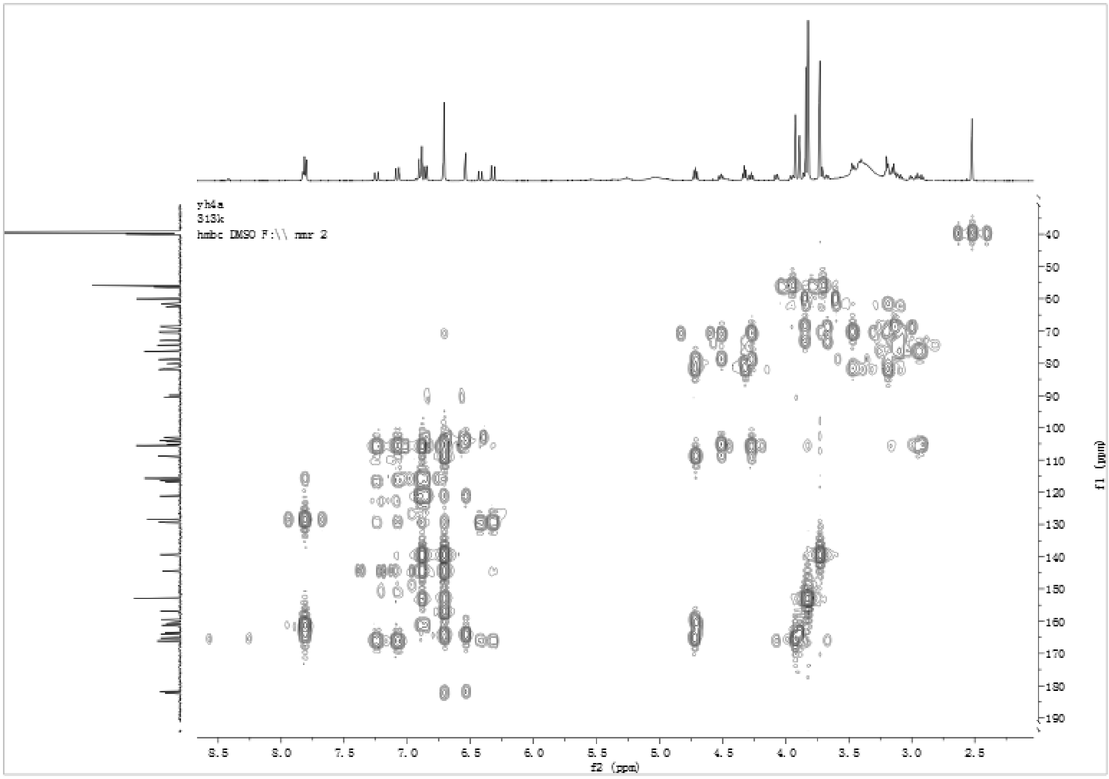

Fig. 12. HREIMS of compound **2**.

Elemental Composition Report **Page 1**

Single Mass Analysis
Tolerance = 10.0 PPM / DBE: min = -10.0, max = 120.0
Selected filters: None

Monoisotopic Mass, Odd and Even Electron Ions
25 formula(e) evaluated with 1 results within limits (up to 51 closest results for each mass)
Elements Used:
C: 0-200 H: 0-400 O: 18-20

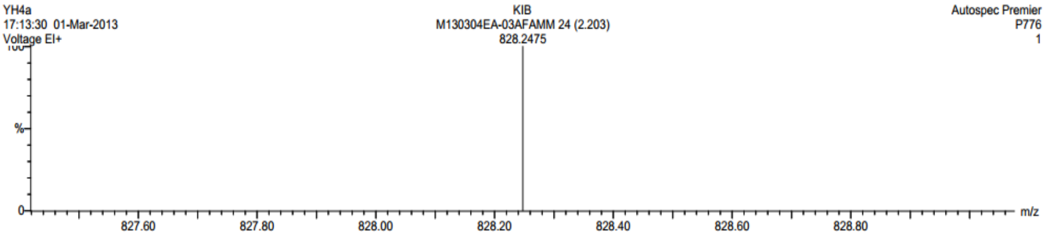

	Mass	Calc. Mass	mDa	PPM	DBE	i-FIT	Formula
Minimum:					-10.0		
Maximum:			200.0	10.0	120.0		
	828.2475	828.2477	-0.2	-0.2	19.0	5546025.5	C40 H44 O19

Fig. 13. ^{1}H NMR (600 MHz, 313 K) spectrum of compound **3** in DMSO- d_6.

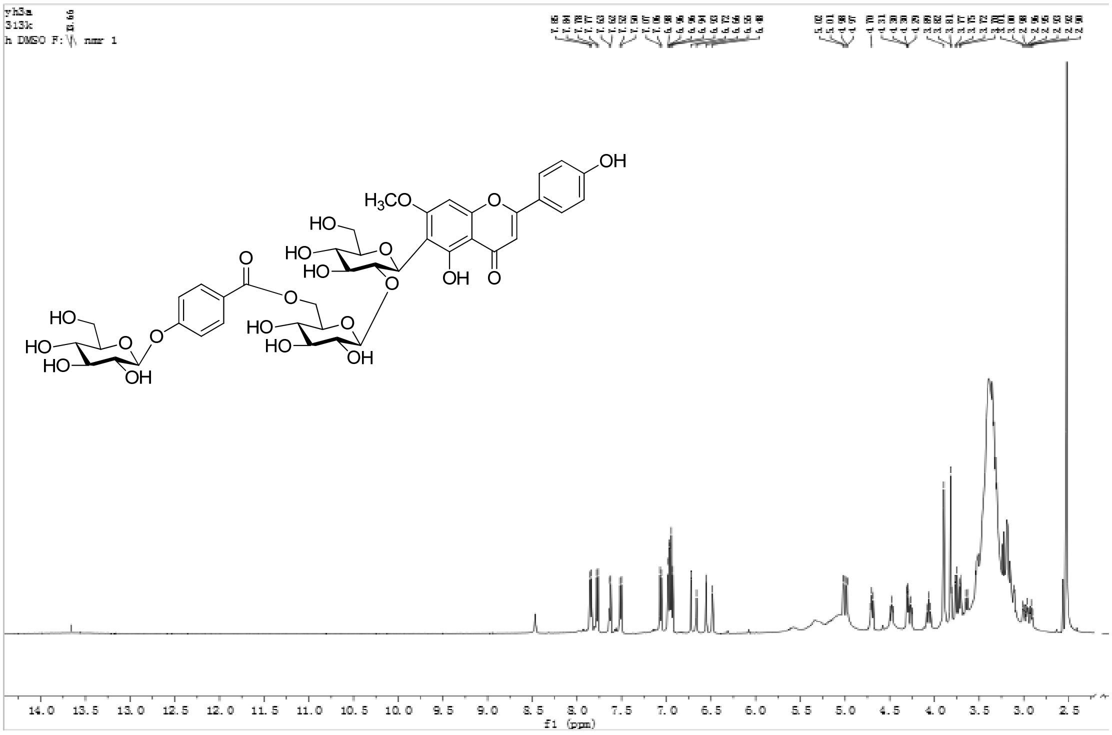

Fig. 14. ^{13}C NMR (150 MHz, 313 K) spectrum of compound **3** in DMSO- d_6.

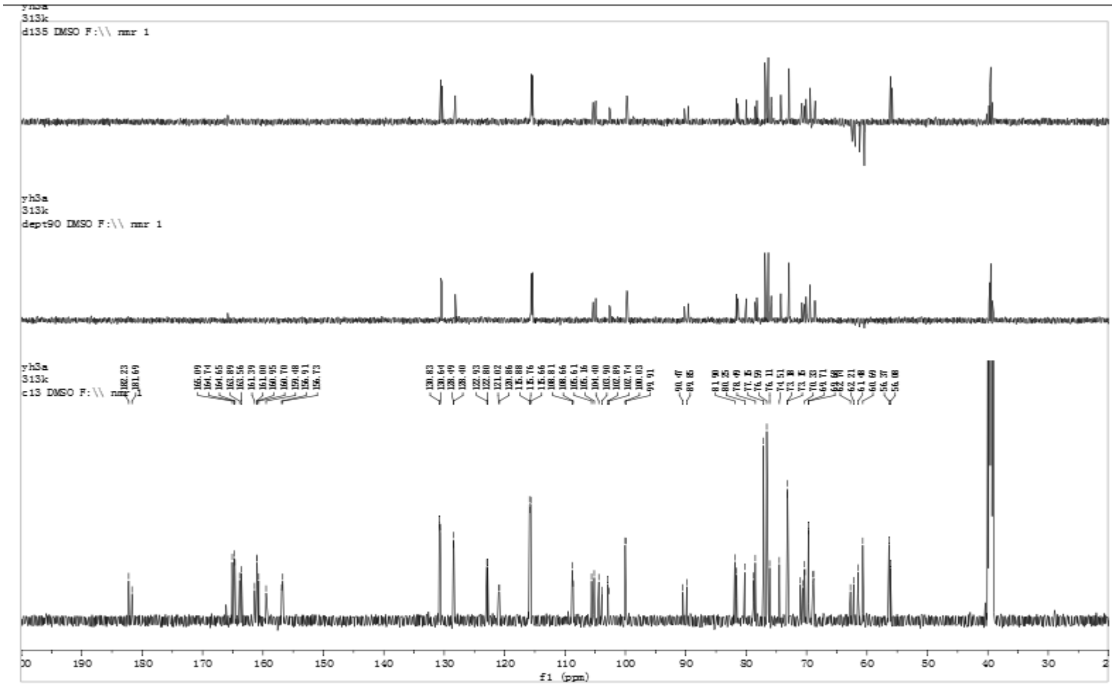

Fig. 15. HSQC (313 K) spectrum of compound **3** in DMSO- d_6.

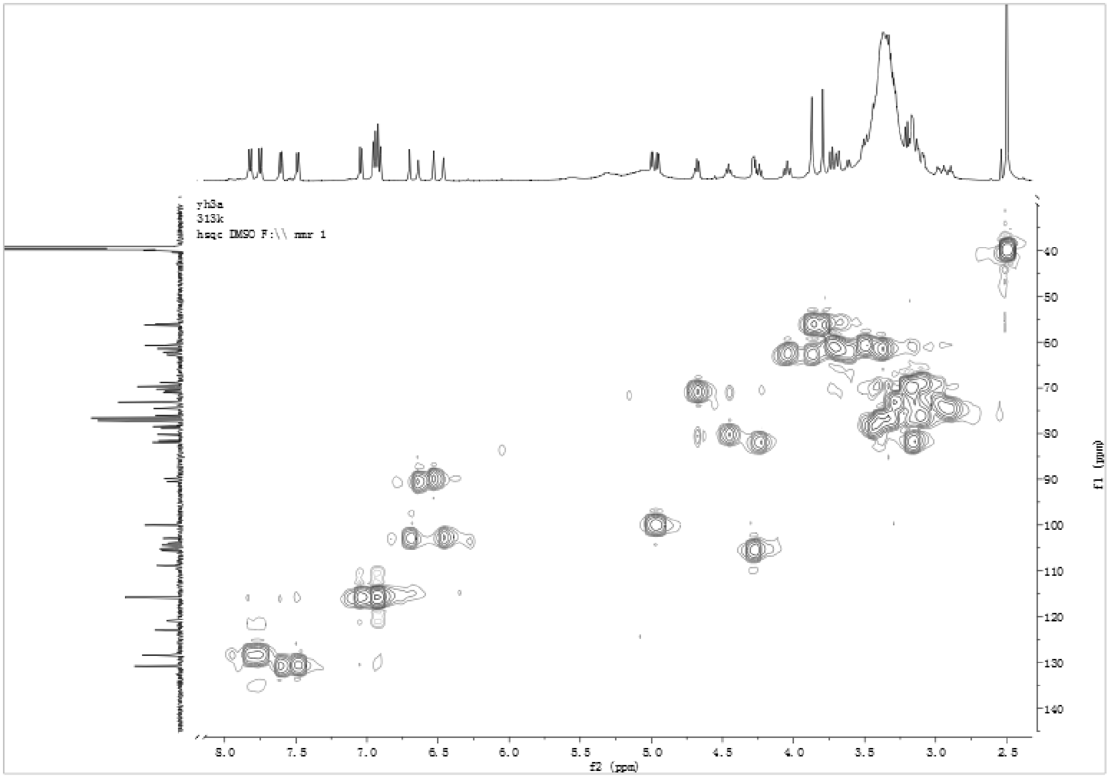

Fig. 16. HMBC (313 K) spectrum of compound **3** in DMSO-d_6.

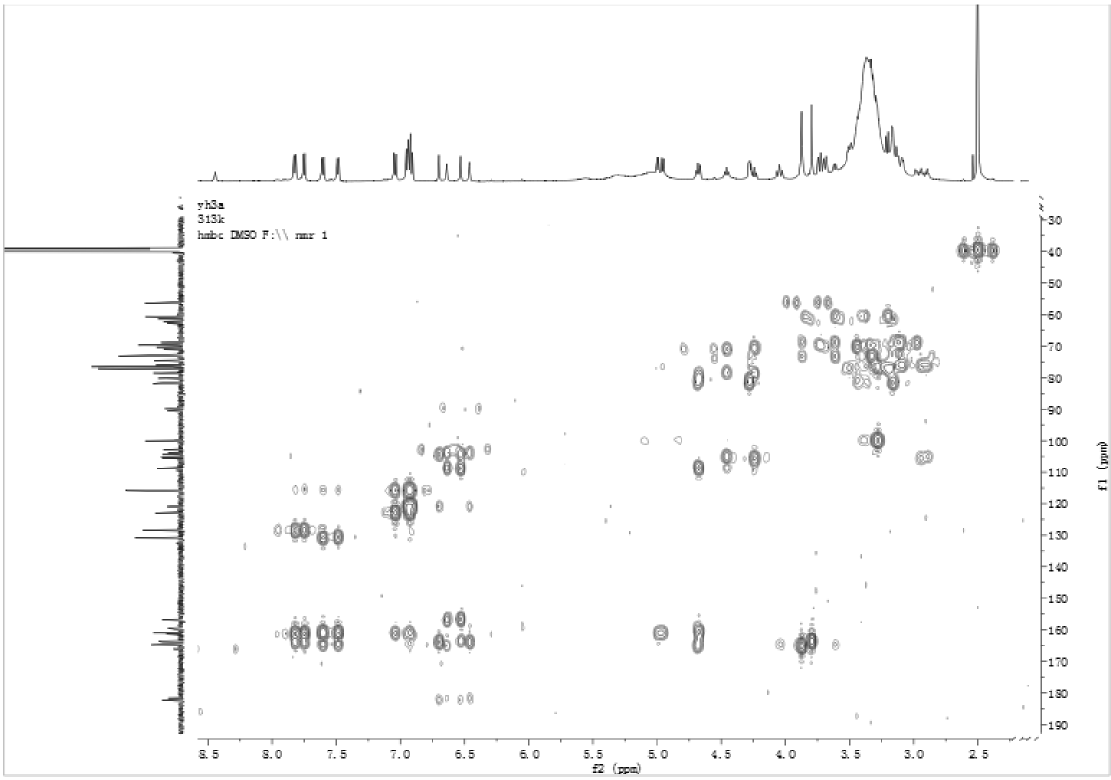

Fig. 17. HREIMS of compound **3**.

Elemental Composition Report **Page 1**

Single Mass Analysis
Tolerance = 10.0 PPM / DBE: min = -10.0, max = 120.0
Selected filters: None

Monoisotopic Mass, Odd and Even Electron Ions
25 formula(e) evaluated with 1 results within limits (up to 51 closest results for each mass)
Elements Used:
C: 0-200 H: 0-400 O: 21-23

YH3a
17:06:33 01-Mar-2013
Voltage EI+

KIB
M130304EA-02AFAMMA 25 (2.294)
890.2523

Autospec Premier
P776
1.67

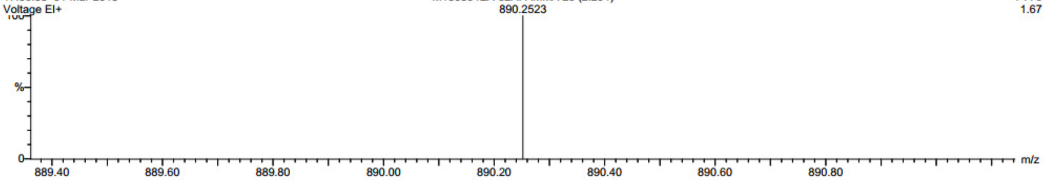

Minimum:				-10.0		
Maximum:		200.0	10.0	120.0		
Mass	Calc. Mass	mDa	PPM	DBE	i-FIT	Formula
890.2523	890.2481	4.2	4.7	19.0	5546025.5	C41 H46 O22

Fig. 18. Chemical structures of known compounds 4–22

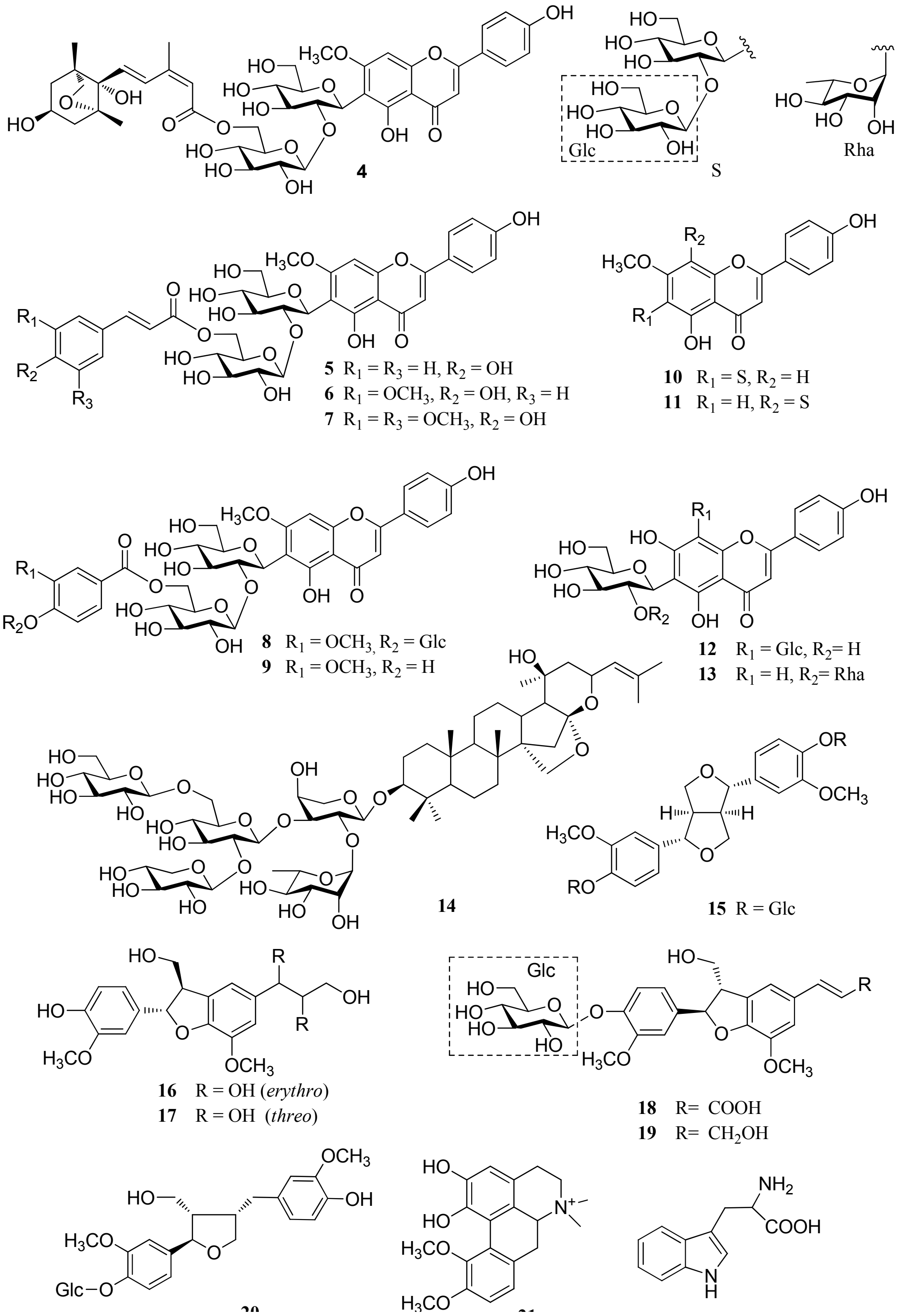

Supplement: Supplementary file 1 — Supplementary material, approximately 846 KB. [file 13659_2013_28_MOESM1_ESM.pdf]
